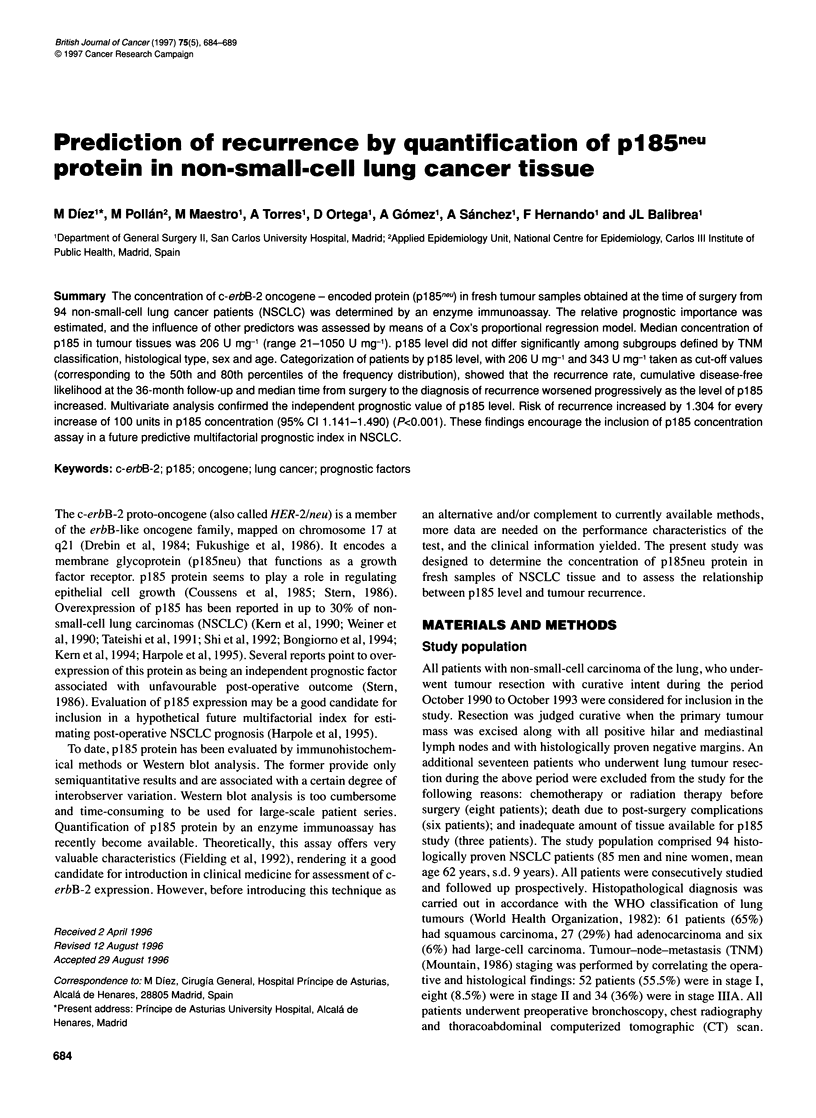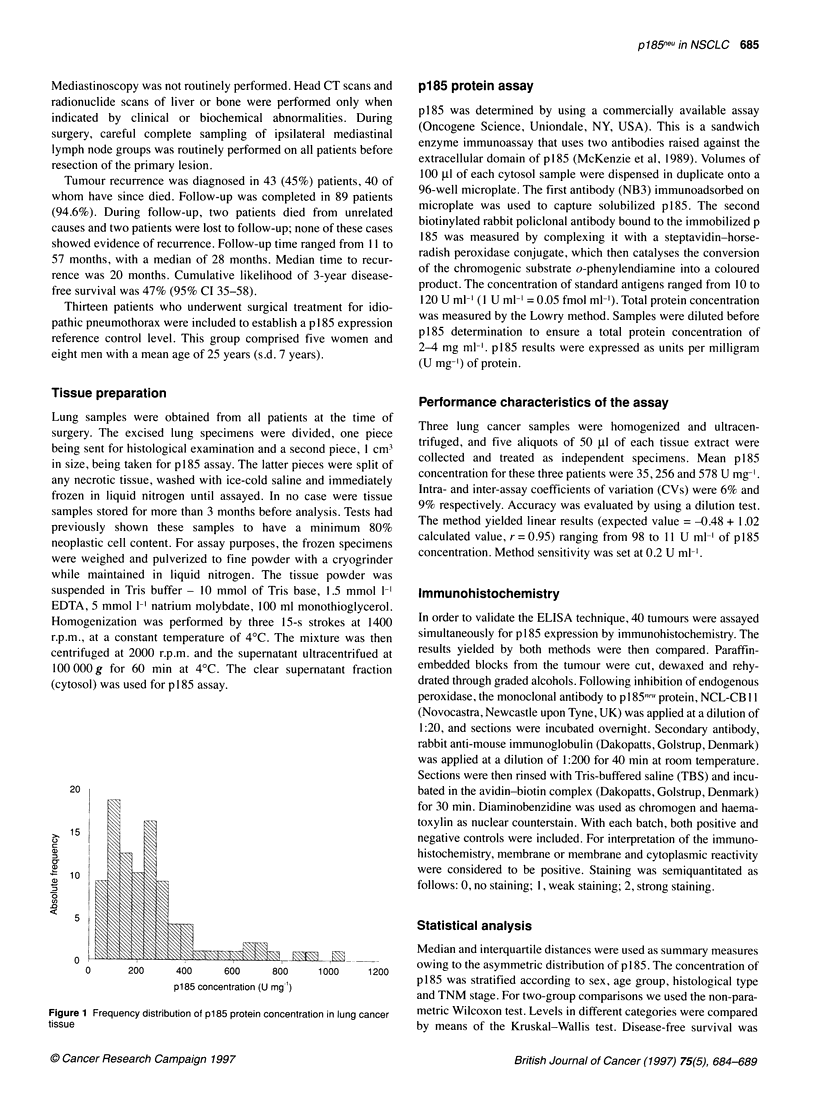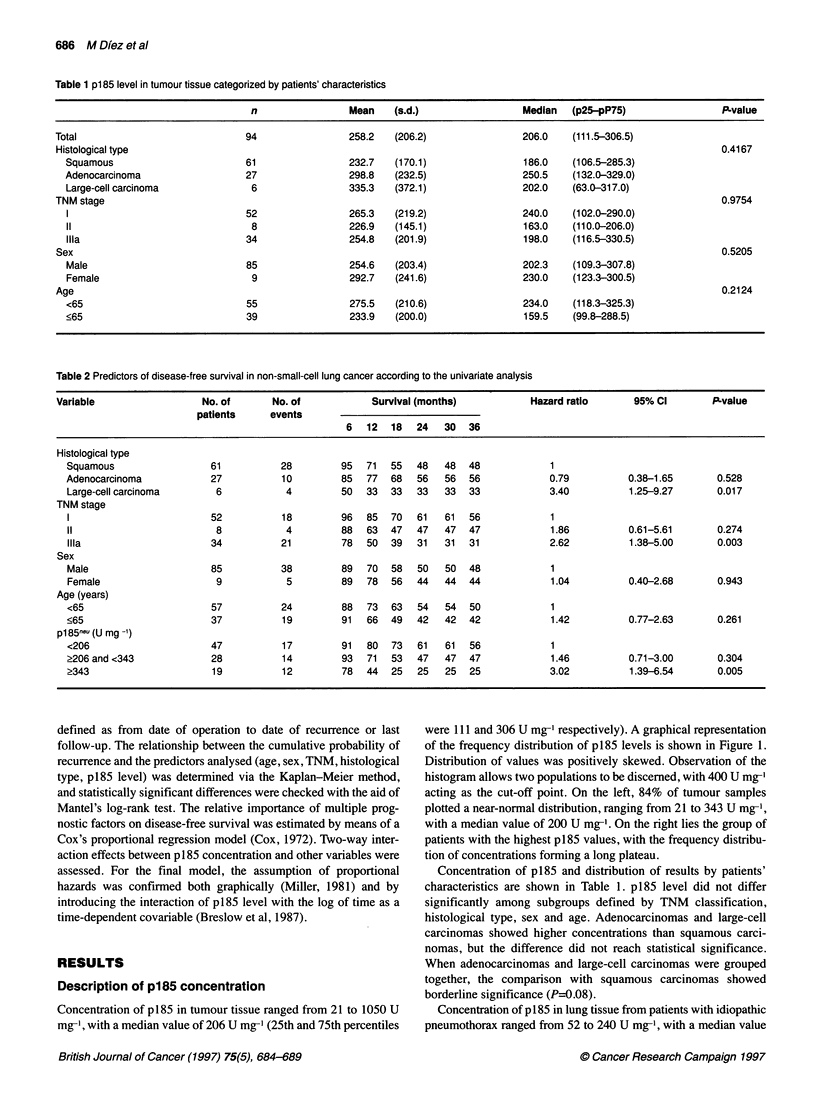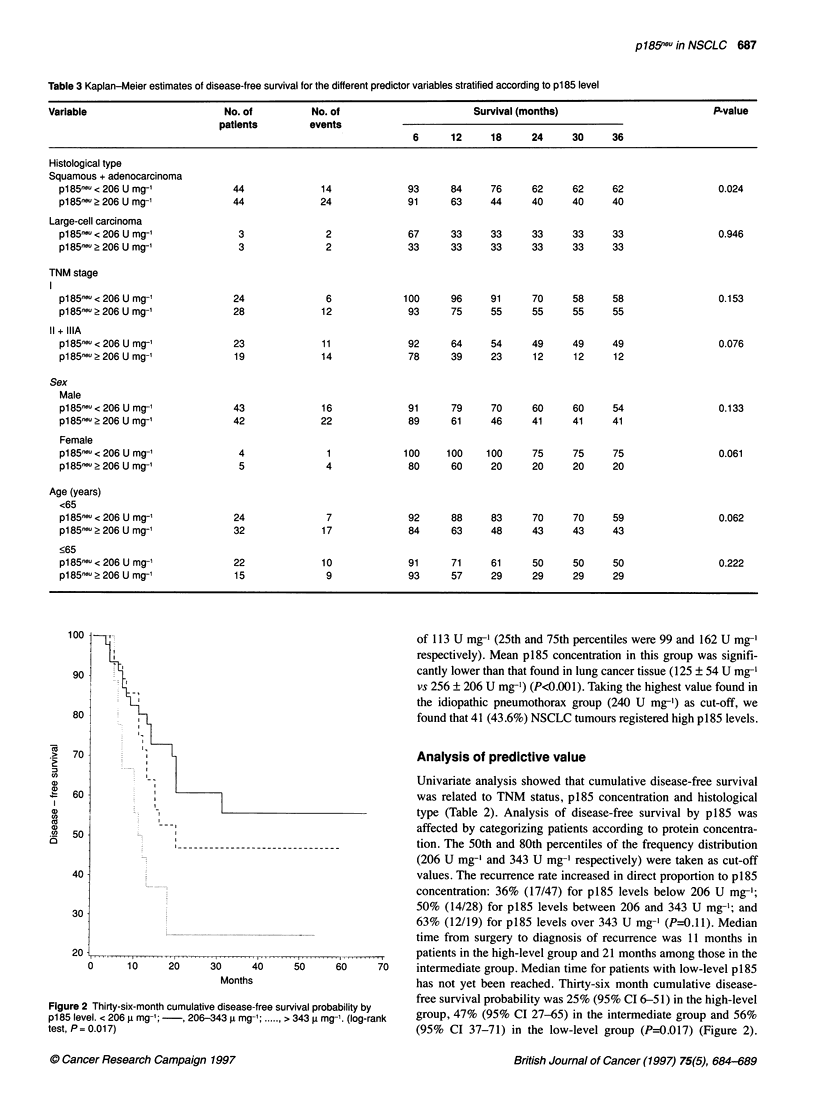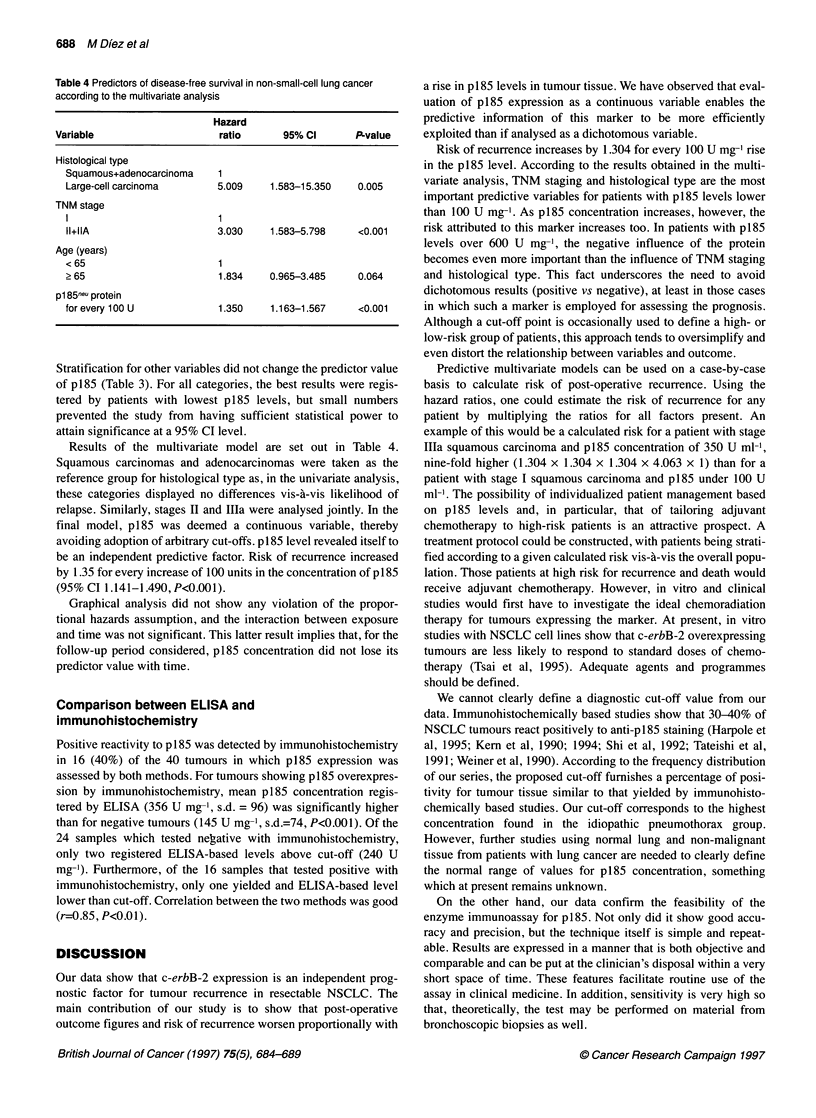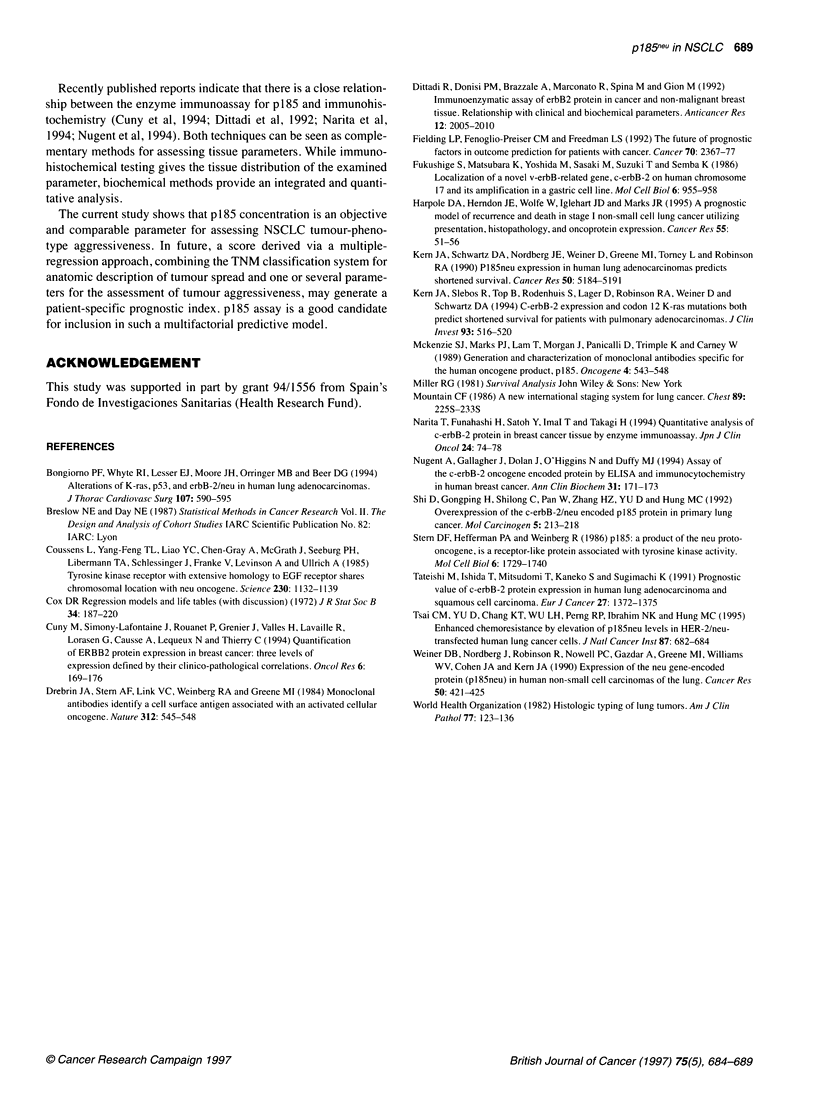# Prediction of recurrence by quantification of p185neu protein in non-small-cell lung cancer tissue.

**DOI:** 10.1038/bjc.1997.122

**Published:** 1997

**Authors:** M. Diez, M. Pollán, M. Maestro, A. Torres, D. Ortega, A. Gómez, A. Sánchez, F. Hernando, J. L. Balibrea

**Affiliations:** Department of General Surgery II, San Carlos University Hospital, Madrid, Spain.

## Abstract

The concentration of c-erbB-2 oncogene-encoded protein (p185neu) in fresh tumour samples obtained at the time of surgery from 94 non-small-cell lung cancer patients (NSCLC) was determined by an enzyme immunoassay. The relative prognostic importance was estimated, and the influence of other predictors was assessed by means of a Cox's proportional regression model. Median concentration of p185 in tumour tissues was 206 U mg(-1) (range 21-1050 U mg(-1)). p185 level did not differ significantly among subgroups defined by TNM classification, histological type, sex and age. Categorization of patients by p185 level, with 206 U mg(-1) and 343 U mg(-1) taken as cut-off values (corresponding to the 50th and 80th percentiles of the frequency distribution), showed that the recurrence rate, cumulative disease-free likelihood at the 36-month follow-up and median time from surgery to the diagnosis of recurrence worsened progressively as the level of p185 increased. Multivariate analysis confirmed the independent prognostic value of p185 level. Risk of recurrence increased by 1.304 for every increase of 100 units in p185 concentration (95% CI 1.141-1.490) (P<0.001). These findings encourage the inclusion of p185 concentration assay in a future predictive multifactorial prognostic index in NSCLC.


British Journal of Cancer (1997) 75(5), 684–689
© 1997 Cancer Research Campaign

### Prediction of recurrence by quantification of p185$^{neu}$ protein in non-small-cell lung cancer tissue

M Díez[1]*, M Pollán[2], M Maestro[1], A Torres[1], D Ortega[1], A Gómez[1], A Sánchez[1], F Hernando[1] and JL Balibrea[1]

[1]Department of General Surgery II, San Carlos University Hospital, Madrid; [2]Applied Epidemiology Unit, National Centre for Epidemiology, Carlos III Institute of Public Health, Madrid, Spain

**Summary** The concentration of c-*erb*B-2 oncogene – encoded protein (p185$^{neu}$) in fresh tumour samples obtained at the time of surgery from 94 non-small-cell lung cancer patients (NSCLC) was determined by an enzyme immunoassay. The relative prognostic importance was estimated, and the influence of other predictors was assessed by means of a Cox's proportional regression model. Median concentration of p185 in tumour tissues was 206 U mg$^{-1}$ (range 21–1050 U mg$^{-1}$). p185 level did not differ significantly among subgroups defined by TNM classification, histological type, sex and age. Categorization of patients by p185 level, with 206 U mg$^{-1}$ and 343 U mg$^{-1}$ taken as cut-off values (corresponding to the 50th and 80th percentiles of the frequency distribution), showed that the recurrence rate, cumulative disease-free likelihood at the 36-month follow-up and median time from surgery to the diagnosis of recurrence worsened progressively as the level of p185 increased. Multivariate analysis confirmed the independent prognostic value of p185 level. Risk of recurrence increased by 1.304 for every increase of 100 units in p185 concentration (95% CI 1.141–1.490) ($P$<0.001). These findings encourage the inclusion of p185 concentration assay in a future predictive multifactorial prognostic index in NSCLC.

**Keywords:** c-*erb*B-2; p185; oncogene; lung cancer; prognostic factors

The c-*erb*B-2 proto-oncogene (also called *HER-2/neu*) is a member of the *erb*B-like oncogene family, mapped on chromosome 17 at q21 (Drebin et al, 1984; Fukushige et al, 1986). It encodes a membrane glycoprotein (p185neu) that functions as a growth factor receptor. p185 protein seems to play a role in regulating epithelial cell growth (Coussens et al, 1985; Stern, 1986). Overexpression of p185 has been reported in up to 30% of non-small-cell lung carcinomas (NSCLC) (Kern et al, 1990; Weiner et al, 1990; Tateishi et al, 1991; Shi et al, 1992; Bongiorno et al, 1994; Kern et al, 1994; Harpole et al, 1995). Several reports point to overexpression of this protein as being an independent prognostic factor associated with unfavourable post-operative outcome (Stern, 1986). Evaluation of p185 expression may be a good candidate for inclusion in a hypothetical future multifactorial index for estimating post-operative NSCLC prognosis (Harpole et al, 1995).

To date, p185 protein has been evaluated by immunohistochemical methods or Western blot analysis. The former provide only semiquantitative results and are associated with a certain degree of interobserver variation. Western blot analysis is too cumbersome and time-consuming to be used for large-scale patient series. Quantification of p185 protein by an enzyme immunoassay has recently become available. Theoretically, this assay offers very valuable characteristics (Fielding et al, 1992), rendering it a good candidate for introduction in clinical medicine for assessment of c-*erb*B-2 expression. However, before introducing this technique as an alternative and/or complement to currently available methods, more data are needed on the performance characteristics of the test, and the clinical information yielded. The present study was designed to determine the concentration of p185neu protein in fresh samples of NSCLC tissue and to assess the relationship between p185 level and tumour recurrence.

### MATERIALS AND METHODS

### Study population

All patients with non-small-cell carcinoma of the lung, who underwent tumour resection with curative intent during the period October 1990 to October 1993 were considered for inclusion in the study. Resection was judged curative when the primary tumour mass was excised along with all positive hilar and mediastinal lymph nodes and with histologically proven negative margins. An additional seventeen patients who underwent lung tumour resection during the above period were excluded from the study for the following reasons: chemotherapy or radiation therapy before surgery (eight patients); death due to post-surgery complications (six patients); and inadequate amount of tissue available for p185 study (three patients). The study population comprised 94 histologically proven NSCLC patients (85 men and nine women, mean age 62 years, s.d. 9 years). All patients were consecutively studied and followed up prospectively. Histopathological diagnosis was carried out in accordance with the WHO classification of lung tumours (World Health Organization, 1982): 61 patients (65%) had squamous carcinoma, 27 (29%) had adenocarcinoma and six (6%) had large-cell carcinoma. Tumour–node–metastasis (TNM) (Mountain, 1986) staging was performed by correlating the operative and histological findings: 52 patients (55.5%) were in stage I, eight (8.5%) were in stage II and 34 (36%) were in stage IIIA. All patients underwent preoperative bronchoscopy, chest radiography and thoracoabdominal computerized tomographic (CT) scan.

*Received 2 April 1996*
*Revised 12 August 1996*
*Accepted 29 August 1996*

*Correspondence to:* M Díez, Cirugía General, Hospital Príncipe de Asturias, Alcalá de Henares, 28805 Madrid, Spain
*Present address: Príncipe de Asturias University Hospital, Alcalá de Henares, Madrid

Mediastinoscopy was not routinely performed. Head CT scans and radionuclide scans of liver or bone were performed only when indicated by clinical or biochemical abnormalities. During surgery, careful complete sampling of ipsilateral mediastinal lymph node groups was routinely performed on all patients before resection of the primary lesion.

Tumour recurrence was diagnosed in 43 (45%) patients, 40 of whom have since died. Follow-up was completed in 89 patients (94.6%). During follow-up, two patients died from unrelated causes and two patients were lost to follow-up; none of these cases showed evidence of recurrence. Follow-up time ranged from 11 to 57 months, with a median of 28 months. Median time to recurrence was 20 months. Cumulative likelihood of 3-year disease-free survival was 47% (95% CI 35–58).

Thirteen patients who underwent surgical treatment for idiopathic pneumothorax were included to establish a p185 expression reference control level. This group comprised five women and eight men with a mean age of 25 years (s.d. 7 years).

### Tissue preparation

Lung samples were obtained from all patients at the time of surgery. The excised lung specimens were divided, one piece being sent for histological examination and a second piece, 1 $cm^3$ in size, being taken for p185 assay. The latter pieces were split of any necrotic tissue, washed with ice-cold saline and immediately frozen in liquid nitrogen until assayed. In no case were tissue samples stored for more than 3 months before analysis. Tests had previously shown these samples to have a minimum 80% neoplastic cell content. For assay purposes, the frozen specimens were weighed and pulverized to fine powder with a cryogrinder while maintained in liquid nitrogen. The tissue powder was suspended in Tris buffer – 10 mmol of Tris base, 1.5 mmol $l^{-1}$ EDTA, 5 mmol $l^{-1}$ natrium molybdate, 100 ml monothioglycerol. Homogenization was performed by three 15-s strokes at 1400 r.p.m., at a constant temperature of 4°C. The mixture was then centrifuged at 2000 r.p.m. and the supernatant ultracentrifued at 100 000 *g* for 60 min at 4°C. The clear supernatant fraction (cytosol) was used for p185 assay.

**Figure 1** Frequency distribution of p185 protein concentration in lung cancer tissue

### p185 protein assay p185 was determined by using a commercially available assay (Oncogene Science, Uniondale, NY, USA). This is a sandwich enzyme immunoassay that uses two antibodies raised against the extracellular domain of p185 (McKenzie et al, 1989). Volumes of 100 μl of each cytosol sample were dispensed in duplicate onto a 96-well microplate. The first antibody (NB3) immunoadsorbed on microplate was used to capture solubilized p185. The second biotinylated rabbit policlonal antibody bound to the immobilized p 185 was measured by complexing it with a steptavidin–horseradish peroxidase conjugate, which then catalyses the conversion of the chromogenic substrate *o*-phenylendiamine into a coloured product. The concentration of standard antigens ranged from 10 to 120 U $ml^{-1}$ (1 U $ml^{-1}$ = 0.05 fmol $ml^{-1}$). Total protein concentration was measured by the Lowry method. Samples were diluted before p185 determination to ensure a total protein concentration of 2–4 mg $ml^{-1}$. p185 results were expressed as units per milligram (U $mg^{-1}$) of protein.

### Performance characteristics of the assay

Three lung cancer samples were homogenized and ultracentrifuged, and five aliquots of 50 μl of each tissue extract were collected and treated as independent specimens. Mean p185 concentration for these three patients were 35, 256 and 578 U $mg^{-1}$. Intra- and inter-assay coefficients of variation (CVs) were 6% and 9% respectively. Accuracy was evaluated by using a dilution test. The method yielded linear results (expected value = –0.48 + 1.02 calculated value, $r = 0.95$) ranging from 98 to 11 U $ml^{-1}$ of p185 concentration. Method sensitivity was set at 0.2 U $ml^{-1}$.

### Immunohistochemistry

In order to validate the ELISA technique, 40 tumours were assayed simultaneously for p185 expression by immunohistochemistry. The results yielded by both methods were then compared. Paraffin-embedded blocks from the tumour were cut, dewaxed and rehydrated through graded alcohols. Following inhibition of endogenous peroxidase, the monoclonal antibody to $p185^{neu}$ protein, NCL-CB11 (Novocastra, Newcastle upon Tyne, UK) was applied at a dilution of 1:20, and sections were incubated overnight. Secondary antibody, rabbit anti-mouse immunoglobulin (Dakopatts, Golstrup, Denmark) was applied at a dilution of 1:200 for 40 min at room temperature. Sections were then rinsed with Tris-buffered saline (TBS) and incubated in the avidin–biotin complex (Dakopatts, Golstrup, Denmark) for 30 min. Diaminobenzidine was used as chromogen and haematoxylin as nuclear counterstain. With each batch, both positive and negative controls were included. For interpretation of the immunohistochemistry, membrane or membrane and cytoplasmic reactivity were considered to be positive. Staining was semiquantitated as follows: 0, no staining; 1, weak staining; 2, strong staining.

### Statistical analysis

Median and interquartile distances were used as summary measures owing to the asymmetric distribution of p185. The concentration of p185 was stratified according to sex, age group, histological type and TNM stage. For two-group comparisons we used the non-parametric Wilcoxon test. Levels in different categories were compared by means of the Kruskal–Wallis test. Disease-free survival was

© Cancer Research Campaign 1997

**Table 1** p185 level in tumour tissue categorized by patients' characteristics

| | n | Mean | (s.d.) | Median | (p25–pP75) | P-value |
|---|---|---|---|---|---|---|
| Total | 94 | 258.2 | (206.2) | 206.0 | (111.5–306.5) | |
| Histological type | | | | | | 0.4167 |
| Squamous | 61 | 232.7 | (170.1) | 186.0 | (106.5–285.3) | |
| Adenocarcinoma | 27 | 298.8 | (232.5) | 250.5 | (132.0–329.0) | |
| Large-cell carcinoma | 6 | 335.3 | (372.1) | 202.0 | (63.0–317.0) | |
| TNM stage | | | | | | 0.9754 |
| I | 52 | 265.3 | (219.2) | 240.0 | (102.0–290.0) | |
| II | 8 | 226.9 | (145.1) | 163.0 | (110.0–206.0) | |
| IIIa | 34 | 254.8 | (201.9) | 198.0 | (116.5–330.5) | |
| Sex | | | | | | 0.5205 |
| Male | 85 | 254.6 | (203.4) | 202.3 | (109.3–307.8) | |
| Female | 9 | 292.7 | (241.6) | 230.0 | (123.3–300.5) | |
| Age | | | | | | 0.2124 |
| <65 | 55 | 275.5 | (210.6) | 234.0 | (118.3–325.3) | |
| ≤65 | 39 | 233.9 | (200.0) | 159.5 | (99.8–288.5) | |

**Table 2** Predictors of disease-free survival in non-small-cell lung cancer according to the univariate analysis

| Variable | No. of patients | No. of events | Survival (months) | | | | | | Hazard ratio | 95% CI | P-value |
|---|---|---|---|---|---|---|---|---|---|---|---|
| | | | 6 | 12 | 18 | 24 | 30 | 36 | | | |
| Histological type | | | | | | | | | | | |
| Squamous | 61 | 28 | 95 | 71 | 55 | 48 | 48 | 48 | 1 | | |
| Adenocarcinoma | 27 | 10 | 85 | 77 | 68 | 56 | 56 | 56 | 0.79 | 0.38–1.65 | 0.528 |
| Large-cell carcinoma | 6 | 4 | 50 | 33 | 33 | 33 | 33 | 33 | 3.40 | 1.25–9.27 | 0.017 |
| TNM stage | | | | | | | | | | | |
| I | 52 | 18 | 96 | 85 | 70 | 61 | 61 | 56 | 1 | | |
| II | 8 | 4 | 88 | 63 | 47 | 47 | 47 | 47 | 1.86 | 0.61–5.61 | 0.274 |
| IIIa | 34 | 21 | 78 | 50 | 39 | 31 | 31 | 31 | 2.62 | 1.38–5.00 | 0.003 |
| Sex | | | | | | | | | | | |
| Male | 85 | 38 | 89 | 70 | 58 | 50 | 50 | 48 | 1 | | |
| Female | 9 | 5 | 89 | 78 | 56 | 44 | 44 | 44 | 1.04 | 0.40–2.68 | 0.943 |
| Age (years) | | | | | | | | | | | |
| <65 | 57 | 24 | 88 | 73 | 63 | 54 | 54 | 50 | 1 | | |
| ≤65 | 37 | 19 | 91 | 66 | 49 | 42 | 42 | 42 | 1.42 | 0.77–2.63 | 0.261 |
| $p185^{neu}$ (U $mg^{-1}$) | | | | | | | | | | | |
| <206 | 47 | 17 | 91 | 80 | 73 | 61 | 61 | 56 | 1 | | |
| ≥206 and <343 | 28 | 14 | 93 | 71 | 53 | 47 | 47 | 47 | 1.46 | 0.71–3.00 | 0.304 |
| ≥343 | 19 | 12 | 78 | 44 | 25 | 25 | 25 | 25 | 3.02 | 1.39–6.54 | 0.005 |

defined as from date of operation to date of recurrence or last follow-up. The relationship between the cumulative probability of recurrence and the predictors analysed (age, sex, TNM, histological type, p185 level) was determined via the Kaplan–Meier method, and statistically significant differences were checked with the aid of Mantel's log-rank test. The relative importance of multiple prognostic factors on disease-free survival was estimated by means of a Cox's proportional regression model (Cox, 1972). Two-way interaction effects between p185 concentration and other variables were assessed. For the final model, the assumption of proportional hazards was confirmed both graphically (Miller, 1981) and by introducing the interaction of p185 level with the log of time as a time-dependent covariable (Breslow et al, 1987).

### RESULTS

### Description of p185 concentration

Concentration of p185 in tumour tissue ranged from 21 to 1050 U $mg^{-1}$, with a median value of 206 U $mg^{-1}$ (25th and 75th percentiles were 111 and 306 U $mg^{-1}$ respectively). A graphical representation of the frequency distribution of p185 levels is shown in Figure 1. Distribution of values was positively skewed. Observation of the histogram allows two populations to be discerned, with 400 U $mg^{-1}$ acting as the cut-off point. On the left, 84% of tumour samples plotted a near-normal distribution, ranging from 21 to 343 U $mg^{-1}$, with a median value of 200 U $mg^{-1}$. On the right lies the group of patients with the highest p185 values, with the frequency distribution of concentrations forming a long plateau.

Concentration of p185 and distribution of results by patients' characteristics are shown in Table 1. p185 level did not differ significantly among subgroups defined by TNM classification, histological type, sex and age. Adenocarcinomas and large-cell carcinomas showed higher concentrations than squamous carcinomas, but the difference did not reach statistical significance. When adenocarcinomas and large-cell carcinomas were grouped together, the comparison with squamous carcinomas showed borderline significance ($P$=0.08).

Concentration of p185 in lung tissue from patients with idiopathic pneumothorax ranged from 52 to 240 U $mg^{-1}$, with a median value

© Cancer Research Campaign 1997

**Table 3** Kaplan–Meier estimates of disease-free survival for the different predictor variables stratified according to p185 level

| Variable | No. of patients | No. of events | Survival (months) | | | | | | *P*-value |
|---|---|---|---|---|---|---|---|---|---|
| | | | 6 | 12 | 18 | 24 | 30 | 36 | |
| Histological type | | | | | | | | | |
| Squamous + adenocarcinoma | | | | | | | | | |
| p185$^{neu}$ < 206 U mg$^{-1}$ | 44 | 14 | 93 | 84 | 76 | 62 | 62 | 62 | 0.024 |
| p185$^{neu}$ ≥ 206 U mg$^{-1}$ | 44 | 24 | 91 | 63 | 44 | 40 | 40 | 40 | |
| Large-cell carcinoma | | | | | | | | | |
| p185$^{neu}$ < 206 U mg$^{-1}$ | 3 | 2 | 67 | 33 | 33 | 33 | 33 | 33 | 0.946 |
| p185$^{neu}$ ≥ 206 U mg$^{-1}$ | 3 | 2 | 33 | 33 | 33 | 33 | 33 | 33 | |
| TNM stage | | | | | | | | | |
| I | | | | | | | | | |
| p185$^{neu}$ < 206 U mg$^{-1}$ | 24 | 6 | 100 | 96 | 91 | 70 | 58 | 58 | 0.153 |
| p185$^{neu}$ ≥ 206 U mg$^{-1}$ | 28 | 12 | 93 | 75 | 55 | 55 | 55 | 55 | |
| II + IIIA | | | | | | | | | |
| p185$^{neu}$ < 206 U mg$^{-1}$ | 23 | 11 | 92 | 64 | 54 | 49 | 49 | 49 | 0.076 |
| p185$^{neu}$ ≥ 206 U mg$^{-1}$ | 19 | 14 | 78 | 39 | 23 | 12 | 12 | 12 | |
| *Sex* | | | | | | | | | |
| Male | | | | | | | | | |
| p185$^{neu}$ < 206 U mg$^{-1}$ | 43 | 16 | 91 | 79 | 70 | 60 | 60 | 54 | 0.133 |
| p185$^{neu}$ ≥ 206 U mg$^{-1}$ | 42 | 22 | 89 | 61 | 46 | 41 | 41 | 41 | |
| Female | | | | | | | | | |
| p185$^{neu}$ < 206 U mg$^{-1}$ | 4 | 1 | 100 | 100 | 100 | 75 | 75 | 75 | 0.061 |
| p185$^{neu}$ ≥ 206 U mg$^{-1}$ | 5 | 4 | 80 | 60 | 20 | 20 | 20 | 20 | |
| Age (years) | | | | | | | | | |
| <65 | | | | | | | | | |
| p185$^{neu}$ < 206 U mg$^{-1}$ | 24 | 7 | 92 | 88 | 83 | 70 | 70 | 59 | 0.062 |
| p185$^{neu}$ ≥ 206 U mg$^{-1}$ | 32 | 17 | 84 | 63 | 48 | 43 | 43 | 43 | |
| ≤65 | | | | | | | | | |
| p185$^{neu}$ < 206 U mg$^{-1}$ | 22 | 10 | 91 | 71 | 61 | 50 | 50 | 50 | 0.222 |
| p185$^{neu}$ ≥ 206 U mg$^{-1}$ | 15 | 9 | 93 | 57 | 29 | 29 | 29 | 29 | |

**Figure 2** Thirty-six-month cumulative disease-free survival probability by p185 level. < 206 μ mg$^{-1}$; ——, 206–343 μ mg$^{-1}$; ....., > 343 μ mg$^{-1}$. (log-rank test, $P = 0.017$)

of 113 U mg$^{-1}$ (25th and 75th percentiles were 99 and 162 U mg$^{-1}$ respectively). Mean p185 concentration in this group was significantly lower than that found in lung cancer tissue (125 ± 54 U mg$^{-1}$ *vs* 256 ± 206 U mg$^{-1}$) ($P$<0.001). Taking the highest value found in the idiopathic pneumothorax group (240 U mg$^{-1}$) as cut-off, we found that 41 (43.6%) NSCLC tumours registered high p185 levels.

### Analysis of predictive value

Univariate analysis showed that cumulative disease-free survival was related to TNM status, p185 concentration and histological type (Table 2). Analysis of disease-free survival by p185 was affected by categorizing patients according to protein concentration. The 50th and 80th percentiles of the frequency distribution (206 U mg$^{-1}$ and 343 U mg$^{-1}$ respectively) were taken as cut-off values. The recurrence rate increased in direct proportion to p185 concentration: 36% (17/47) for p185 levels below 206 U mg$^{-1}$; 50% (14/28) for p185 levels between 206 and 343 U mg$^{-1}$; and 63% (12/19) for p185 levels over 343 U mg$^{-1}$ ($P$=0.11). Median time from surgery to diagnosis of recurrence was 11 months in patients in the high-level group and 21 months among those in the intermediate group. Median time for patients with low-level p185 has not yet been reached. Thirty-six month cumulative disease-free survival probability was 25% (95% CI 6–51) in the high-level group, 47% (95% CI 27–65) in the intermediate group and 56% (95% CI 37–71) in the low-level group ($P$=0.017) (Figure 2).

Table 4 Predictors of disease-free survival in non-small-cell lung cancer according to the multivariate analysis

| Variable | Hazard ratio | 95% CI | P-value |
|---|---|---|---|
| Histological type | | | |
| Squamous+adenocarcinoma | 1 | | |
| Large-cell carcinoma | 5.009 | 1.583–15.350 | 0.005 |
| TNM stage | | | |
| I | 1 | | |
| II+IIA | 3.030 | 1.583–5.798 | <0.001 |
| Age (years) | | | |
| < 65 | 1 | | |
| ≥ 65 | 1.834 | 0.965–3.485 | 0.064 |
| $p185^{neu}$ protein | | | |
| for every 100 U | 1.350 | 1.163–1.567 | <0.001 |

Stratification for other variables did not change the predictor value of p185 (Table 3). For all categories, the best results were registered by patients with lowest p185 levels, but small numbers prevented the study from having sufficient statistical power to attain significance at a 95% CI level.

Results of the multivariate model are set out in Table 4. Squamous carcinomas and adenocarcinomas were taken as the reference group for histological type as, in the univariate analysis, these categories displayed no differences vis-à-vis likelihood of relapse. Similarly, stages II and IIIa were analysed jointly. In the final model, p185 was deemed a continuous variable, thereby avoiding adoption of arbitrary cut-offs. p185 level revealed itself to be an independent predictive factor. Risk of recurrence increased by 1.35 for every increase of 100 units in the concentration of p185 (95% CI 1.141–1.490, $P$<0.001).

Graphical analysis did not show any violation of the proportional hazards assumption, and the interaction between exposure and time was not significant. This latter result implies that, for the follow-up period considered, p185 concentration did not lose its predictor value with time.

### Comparison between ELISA and immunohistochemistry

Positive reactivity to p185 was detected by immunohistochemistry in 16 (40%) of the 40 tumours in which p185 expression was assessed by both methods. For tumours showing p185 overexpression by immunohistochemistry, mean p185 concentration registered by ELISA (356 U $mg^{-1}$, s.d. = 96) was significantly higher than for negative tumours (145 U $mg^{-1}$, s.d.=74, $P$<0.001). Of the 24 samples which tested negative with immunohistochemistry, only two registered ELISA-based levels above cut-off (240 U $mg^{-1}$). Furthermore, of the 16 samples that tested positive with immunohistochemistry, only one yielded and ELISA-based level lower than cut-off. Correlation between the two methods was good ($r$=0.85, $P$<0.01).

### DISCUSSION

Our data show that c-*erb*B-2 expression is an independent prognostic factor for tumour recurrence in resectable NSCLC. The main contribution of our study is to show that post-operative outcome figures and risk of recurrence worsen proportionally with a rise in p185 levels in tumour tissue. We have observed that evaluation of p185 expression as a continuous variable enables the predictive information of this marker to be more efficiently exploited than if analysed as a dichotomous variable.

Risk of recurrence increases by 1.304 for every 100 U $mg^{-1}$ rise in the p185 level. According to the results obtained in the multivariate analysis, TNM staging and histological type are the most important predictive variables for patients with p185 levels lower than 100 U $mg^{-1}$. As p185 concentration increases, however, the risk attributed to this marker increases too. In patients with p185 levels over 600 U $mg^{-1}$, the negative influence of the protein becomes even more important than the influence of TNM staging and histological type. This fact underscores the need to avoid dichotomous results (positive *vs* negative), at least in those cases in which such a marker is employed for assessing the prognosis. Although a cut-off point is occasionally used to define a high- or low-risk group of patients, this approach tends to oversimplify and even distort the relationship between variables and outcome.

Predictive multivariate models can be used on a case-by-case basis to calculate risk of post-operative recurrence. Using the hazard ratios, one could estimate the risk of recurrence for any patient by multiplying the ratios for all factors present. An example of this would be a calculated risk for a patient with stage IIIa squamous carcinoma and p185 concentration of 350 U $ml^{-1}$, nine-fold higher ($1.304 \times 1.304 \times 1.304 \times 4.063 \times 1$) than for a patient with stage I squamous carcinoma and p185 under 100 U $ml^{-1}$. The possibility of individualized patient management based on p185 levels and, in particular, that of tailoring adjuvant chemotherapy to high-risk patients is an attractive prospect. A treatment protocol could be constructed, with patients being stratified according to a given calculated risk vis-à-vis the overall population. Those patients at high risk for recurrence and death would receive adjuvant chemotherapy. However, in vitro and clinical studies would first have to investigate the ideal chemoradiation therapy for tumours expressing the marker. At present, in vitro studies with NSCLC cell lines show that c-*erb*B-2 overexpressing tumours are less likely to respond to standard doses of chemotherapy (Tsai et al, 1995). Adequate agents and programmes should be defined.

We cannot clearly define a diagnostic cut-off value from our data. Immunohistochemically based studies show that 30–40% of NSCLC tumours react positively to anti-p185 staining (Harpole et al, 1995; Kern et al, 1990; 1994; Shi et al, 1992; Tateishi et al, 1991; Weiner et al, 1990). According to the frequency distribution of our series, the proposed cut-off furnishes a percentage of positivity for tumour tissue similar to that yielded by immunohistochemically based studies. Our cut-off corresponds to the highest concentration found in the idiopathic pneumothorax group. However, further studies using normal lung and non-malignant tissue from patients with lung cancer are needed to clearly define the normal range of values for p185 concentration, something which at present remains unknown.

On the other hand, our data confirm the feasibility of the enzyme immunoassay for p185. Not only did it show good accuracy and precision, but the technique itself is simple and repeatable. Results are expressed in a manner that is both objective and comparable and can be put at the clinician's disposal within a very short space of time. These features facilitate routine use of the assay in clinical medicine. In addition, sensitivity is very high so that, theoretically, the test may be performed on material from bronchoscopic biopsies as well.

© Cancer Research Campaign 1997

Recently published reports indicate that there is a close relationship between the enzyme immunoassay for p185 and immunohistochemistry (Cuny et al, 1994; Dittadi et al, 1992; Narita et al, 1994; Nugent et al, 1994). Both techniques can be seen as complementary methods for assessing tissue parameters. While immunohistochemical testing gives the tissue distribution of the examined parameter, biochemical methods provide an integrated and quantitative analysis.

The current study shows that p185 concentration is an objective and comparable parameter for assessing NSCLC tumour-phenotype aggressiveness. In future, a score derived via a multiple-regression approach, combining the TNM classification system for anatomic description of tumour spread and one or several parameters for the assessment of tumour aggressiveness, may generate a patient-specific prognostic index. p185 assay is a good candidate for inclusion in such a multifactorial predictive model.

### ACKNOWLEDGEMENT

This study was supported in part by grant 94/1556 from Spain's Fondo de Investigaciones Sanitarias (Health Research Fund).

### REFERENCES

Bongiorno PF, Whyte RI, Lesser EJ, Moore JH, Orringer MB and Beer DG (1994) Alterations of K-ras, p53, and erbB-2/neu in human lung adenocarcinomas. *J Thorac Cardiovasc Surg* **107**: 590–595

Breslow NE and Day NE (1987) *Statistical Methods in Cancer Research* Vol. II. *The Design and Analysis of Cohort Studies* IARC Scientific Publication No. 82: IARC: Lyon

Coussens L, Yang-Feng TL, Liao YC, Chen-Gray A, McGrath J, Seeburg PH, Libermann TA, Schlessinger J, Franke V, Levinson A and Ullrich A (1985) Tyrosine kinase receptor with extensive homology to EGF receptor shares chromosomal location with neu oncogene. *Science* **230**: 1132–1139

Cox DR Regression models and life tables (with discussion) (1972) *J R Stat Soc B* **34**: 187–220

Cuny M, Simony-Lafontaine J, Rouanet P, Grenier J, Valles H, Lavaille R, Lorasen G, Causse A, Lequeux N and Thierry C (1994) Quantification of ERBB2 protein expression in breast cancer: three levels of expression defined by their clinico-pathological correlations. *Oncol Res* **6**: 169–176

Drebrin JA, Stern AF, Link VC, Weinberg RA and Greene MI (1984) Monoclonal antibodies identify a cell surface antigen associated with an activated cellular oncogene. *Nature* **312**: 545–548

Dittadi R, Donisi PM, Brazzale A, Marconato R, Spina M and Gion M (1992) Immunoenzymatic assay of erbB2 protein in cancer and non-malignant breast tissue. Relationship with clinical and biochemical parameters. *Anticancer Res* **12**: 2005–2010

Fielding LP, Fenoglio-Preiser CM and Freedman LS (1992) The future of prognostic factors in outcome prediction for patients with cancer. *Cancer* **70**: 2367–77

Fukushige S, Matsubara K, Yoshida M, Sasaki M, Suzuki T and Semba K (1986) Localization of a novel v-erbB-related gene, c-erbB-2 on human chromosome 17 and its amplification in a gastric cell line. *Mol Cell Biol* **6**: 955–958

Harpole DA, Herndon JE, Wolfe W, Iglehart JD and Marks JR (1995) A prognostic model of recurrence and death in stage I non-small cell lung cancer utilizing presentation, histopathology, and oncoprotein expression. *Cancer Res* **55**: 51–56

Kern JA, Schwartz DA, Nordberg JE, Weiner D, Greene MI, Torney L and Robinson RA (1990) P185neu expression in human lung adenocarcinomas predicts shortened survival. *Cancer Res* **50**: 5184–5191

Kern JA, Slebos R, Top B, Rodenhuis S, Lager D, Robinson RA, Weiner D and Schwartz DA (1994) C-erbB-2 expression and codon 12 K-ras mutations both predict shortened survival for patients with pulmonary adenocarcinomas. *J Clin Invest* **93**: 516–520

Mckenzie SJ, Marks PJ, Lam T, Morgan J, Panicalli D, Trimple K and Carney W (1989) Generation and characterization of monoclonal antibodies specific for the human oncogene product, p185. *Oncogene* **4**: 543–548

Miller RG (1981) *Survival Analysis* John Wiley & Sons: New York

Mountain CF (1986) A new international staging system for lung cancer. *Chest* **89**: 225S–233S

Narita T, Funahashi H, Satoh Y, ImaI T and Takagi H (1994) Quantitative analysis of c-erbB-2 protein in breast cancer tissue by enzyme immunoassay. *Jpn J Clin Oncol* **24**: 74–78

Nugent A, Gallagher J, Dolan J, O'Higgins N and Duffy MJ (1994) Assay of the c-erbB-2 oncogene encoded protein by ELISA and immunocytochemistry in human breast cancer. *Ann Clin Biochem* **31**: 171–173

Shi D, Gongping H, Shilong C, Pan W, Zhang HZ, YU D and Hung MC (1992) Overexpression of the c-erbB-2/neu encoded p185 protein in primary lung cancer. *Mol Carcinogen* **5**: 213–218

Stern DF, Hefferman PA and Weinberg R (1986) p185: a product of the neu proto-oncogene, is a receptor-like protein associated with tyrosine kinase activity. *Mol Cell Biol* **6**: 1729–1740

Tateishi M, Ishida T, Mitsudomi T, Kaneko S and Sugimachi K (1991) Prognostic value of c-erbB-2 protein expression in human lung adenocarcinoma and squamous cell carcinoma. *Eur J Cancer* **27**: 1372–1375

Tsai CM, YU D, Chang KT, WU LH, Perng RP, Ibrahim NK and Hung MC (1995) Enhanced chemoresistance by elevation of p185neu levels in HER-2/neu-transfected human lung cancer cells. *J Natl Cancer Inst* **87**: 682–684

Weiner DB, Nordberg J, Robinson R, Nowell PC, Gazdar A, Greene MI, Williams WV, Cohen JA and Kern JA (1990) Expression of the neu gene-encoded protein (p185neu) in human non-small cell carcinomas of the lung. *Cancer Res* **50**: 421–425

World Health Organization (1982) Histologic typing of lung tumors. *Am J Clin Pathol* **77**: 123–136

© Cancer Research Campaign 1997